# Eleutheroembryo-based new approach methodologies for endocrine and metabolic endpoints: a comment on Christou et al. (2026)^☆^^☆☆^

**DOI:** 10.1016/j.namjnl.2026.100133

**Published:** 2026-09-18

**Authors:** Gregory F. Lemkine, François Brion, Xavier Coumoul, Jean-Baptiste Fini

**Affiliations:** a WatchFrog SA, 4 rue Pierre Fontaine, 91000 Évry-Courcouronnes, France; b Institut National de l’Environnement Industriel et des Risques (INERIS), Rue Jacques Taffanel, Parc Technologique Alata, 60550, Verneuil-en-Halatte, France; c Université Paris Cité, INSERM, Health & Functional Exposomics - HealthFex, 75006, Paris, France; d Muséum National d’Histoire Naturelle, Team “Responses to Environmental Challenges”, 7 rue Cuvier, 75005, Paris, France

**Keywords:** New approach methodologies, Eleutheroembryo assays, Endocrine disruption, OECD test guidelines, Regulatory readiness

Dear Editor-in-Chief,

We would like to congratulate Maria Christou and colleagues on their remarkable and timely review of new approach methodologies (NAMs) for environmental safety assessment of chemicals (Christou et al., 2026). The paper provides a valuable and well-structured literature-based overview of the current status, challenges and strategies for adoption and acceptance of NAMs by researchers and regulators working in the field of chemical risk assessment. The transparency of the search strategy and the public release of the accompanying ATEST database make this a resource of lasting value to the community.

We are writing, however, to raise a point of framing rather than an oversight. The authors state explicitly (Section 2.1) that alternative approaches for assessing endocrine disruption were not the focus of the review, having previously been covered by Mitchell et al. (2023), while noting that the corresponding cases were nevertheless registered in the ATEST database. This is a legitimate and clearly signalled scoping decision, and nothing in this letter should be read as suggesting that the authors overlooked this literature.

We would, however, like to draw attention to a consequence of that decision. Endocrine disruption does not in practice remain outside the narrative of the review. It appears as an endpoint category in Table 2 (96 in vitro and ex vivo cases) and in Fig. 6 for embryo assays; it is identified in the gap analysis (Fig. 7, Section 4.1) as one of the few endpoint categories covered across all reviewed NAMs; and it appears among the key recommendations, where the authors propose that sublethal endpoints covering endocrine disruption (thyroid activity in particular) could be added to fish embryo toxicity tests. Readers are therefore given a partial picture of the endocrine-related NAM landscape, in particular the absence of already-standardised endocrine eleutheroembryo assays from the readiness landscape presented in Table 3. This is noteworthy, because embryo assays have for several years provided mechanistically informative and ethically favourable tools to identify endocrine-disrupting chemicals within NAM-based strategies.

Below we distinguish two categories of eleutheroembryo-based assays, which bear on the review in different ways: those already adopted as OECD Test Guidelines, and those published but still at earlier stages of evaluation.

## 1 Assays adopted as OECD test guidelines

A set of mechanism-based embryo assays has been developed using fluorescent reporters in early life stages considered as alternatives to animal tests (Mitchell et al., 2023). Four of these have been adopted as OECD Test Guidelines:

TG 248 (2019) — Xenopus Eleutheroembryonic Thyroid Assay (XETA), using the Xenopus laevis TH/bZIP-GFP line, originally described by Fini et al. (2007) and subsequently evaluated for regulatory applicability and compared with the Amphibian Metamorphosis Assay by Du Pasquier et al. (2024) (OECD, 2019);

TG 250 (2021) — EASZY assay, based on the transgenic tg(cyp19a1b:GFP) zebrafish line defined at the construct level by Tong et al. (2009) and established as a quantitative in vivo screen for estrogenic chemicals and mixtures by Brion et al. (2012) (OECD, 2021);

TG 251 (2022) — Rapid Androgen Disruption Activity Reporter (RADAR) assay, using the medaka spg1-GFP androgen reporter introduced by Sébillot et al. (2014) (OECD, 2022);

TG 252 (2024) — Rapid Estrogen Activity In Vivo (REACTIV) assay, based on the medaka chgh-GFP estrogen reporter defined by Spirhanzlova et al. (2016) (OECD, 2024).

Additional work on mixture responses and mechanistic interpretation around the cyp19a1b promoter (see e.g. Petersen et al., 2013; Hinfray et al., 2018) further illustrates the mechanistic understanding that these embryo models can provide.

This has a direct bearing on Section 4.2 and Table 3. Under the readiness scheme proposed by the authors themselves, a method "completed and approved by a standardisation organisation" corresponds to NAMRL 8, a level illustrated in Table 3 by OECD 319, the FET test (TG 236) and the RTgill-W1 assay (TG 249). The four Test Guidelines listed above satisfy the same criterion and would, in our reading, sit alongside them. Their inclusion would qualify the statement that the number of environmental NAMs available at higher readiness levels is fairly limited, and would strengthen rather than weaken the authors’ central argument that NAMs can and do reach regulatory standardisation (see e.g. Du Pasquier et al., 2025).

We would add that Mitchell et al. (2023), to which the review defers on this topic, necessarily predates the adoption of TG 252 in June 2024. A reader following that pointer would therefore not obtain a fully current picture of the adopted guidelines.

We are careful not to overstate the status of these assays. They sit at Level 3 of the OECD Conceptual Framework: they provide in vivo mechanistic information on endocrine activity rather than evidence of adverse apical outcomes, and current ECHA/EFSA guidance does not treat them as stand-alone substitutes for the established fish and amphibian endocrine test guidelines. Their applicability domains also remain incomplete — the XETA, for instance, shows limited sensitivity to inhibition of thyroid hormone synthesis at the sodium-iodide symporter (NIS) and thyroperoxidase (TPO) level (Du Pasquier et al., 2024). These assays are not general stand-alone replacements across endocrine modalities, although the XETA can replace the Amphibian Metamorphosis Assay in defined circumstances under current ECHA/EFSA guidance. Their value at present lies in mechanistic screening and in weight-of-evidence approaches, which is precisely the framing the authors advocate elsewhere in the review.

## 2 Published assays at earlier stages of evaluation

More recently, efforts have been made to develop new reporter assays using zebrafish and medaka embryos to evaluate metabolic endocrine disruptors and thyroid disruption. These assays have been published but have not been through interlaboratory validation or standardisation. We cite them not as validated methods, and make no claim as to their regulatory status or to any future validation timeline, but because they address endpoint categories that the review itself identifies as insufficiently covered.

On the thyroid axis, Table 3 places inclusion of thyroid endpoints in OECD fish tests at NAMRL 7, and the key recommendations note that thyroid endpoints for fish embryo tests are currently under development at the OECD level. Consistent with this, reporter assays have been developed in zebrafish and medaka embryos to evaluate thyroid-mediated activity (Jaka et al., 2023; Pesce et al., 2023). Among these, the TETHYS (Transgenic Eleuthero-embryonic THYroid-Specific) assay reports on thyroid hormone synthesis at the level of the thyroid itself, rather than on the transcriptional response to available thyroid hormone as the XETA does; published work with reference chemicals acting through NIS and TPO inhibition indicates sensitivity to mechanisms that fall outside the XETA applicability domain discussed above (Pesce et al., 2024).

On metabolic disruption, Section 4.1 identifies this disruption as one of the endpoint categories with fewer than ten registered cases, and the key recommendations list it among endpoints "not well covered by NAMs" for which assays need to be developed and evaluated. A recent eleutheroembryo-based assay addresses precisely this gap and illustrates how mechanistically anchored embryo models can integrate xenobiotic-sensing and metabolism-related pathways: the EMERGE assay uses a transgenic tg(cyp3a65:GFP) zebrafish line reporting expression of the orthologue of human CYP3A4 in the developing intestine, and screens for metabolic endocrine-disrupting activity mediated by the zebrafish xenosensors zfPXR and zfAhR2 (Erradhouani et al., 2025a, 2025b).

## 3 Concluding remarks

Taken together, these publications show that embryo-based NAMs can move endocrine testing beyond traditional endpoints measured in chronic fish and amphibian tests toward more specific mechanism-oriented readouts in early life stages, aligned with the protection of laboratory animals and 3Rs objectives. Because the review’s gap analysis and readiness framework are likely to be widely cited as a reference picture of where environmental NAMs currently stand, the absence of these assays may leave readers with the impression that eleutheroembryo assays are not yet part of the toolbox for endocrine and metabolic endpoints.

We would also raise one methodological question, offered in the spirit of assisting future updates. The search strategy summarised in Fig. 1 excludes records matching "human" (Keyword 6). Several aquatic NAMs are characterised explicitly by reference to their human orthologues. The EMERGE assay, for example, reports on the zebrafish orthologue of human CYP3A4 and its abstract discusses human relevance throughout. We wonder whether this filter may have inadvertently excluded aquatic assays whose added value lies precisely in cross-species conservation, an objective the authors themselves highlight in their recommendations on cross-species extrapolation.

Our comments are also consistent with the European Commission’s "Roadmap towards phasing out animal testing for chemical safety assessments" (C(2026) 3497) and the accompanying Commission Staff Working Document SWD(2026) 144, both adopted in June 2026 (European Commission, 2026a, 2026b). In its endocrine-disruption section, the Staff Working Document recognises that several NAMs based on fish or amphibian eleutheroembryos — such as the XETA and transgenic fish embryo assays targeting estrogenic and androgenic activity — can provide mechanistically anchored information on thyroid and sex-steroid axes and may contribute to reducing reliance on longer animal tests. At the same time, SWD(2026) 144 notes that current gaps in validated coverage of endocrine-related mechanisms (for example for upstream steroidogenic effects) still limit the extent to which these eleutheroembryo assays can fully substitute established fish endocrine test guidelines. Ongoing research is progressively closing these gaps, thereby further promoting testing strategies with reduced reliance on animal testing.

We would like to emphasise that this comment is offered in a constructive spirit, with the intention of further strengthening the conclusion of Christou et al. (2026) that NAMs are becoming central to next-generation risk assessment. Their review represents an important contribution to the field, and we hope that the authors and the journal may consider integrating the four adopted Test Guidelines alongside the readiness analysis presented in Table 3, either in a corrigendum or by publishing this letter, so that the picture of standardised environmental NAMs is as complete as the review’s systematic approach deserves.

Sincerely,

Gregory F. Lemkine, François Brion, Xavier Coumoul and Jean-Baptiste Fini.

## Funding

This work did not receive any specific grant from funding agencies in the public, commercial, or not-for-profit sectors.

## Declaration of competing interest

Gregory F. Lemkine is Scientific Director of WatchFrog SA, a company that develops and commercialises several of the embryo-based assays discussed in this letter (XETA, RADAR, REACTIV and TETHYS). François Brion contributed to the development of the EASZY and EMERGE assays and was in charge of the validation of the EASZY assay. Xavier Coumoul is a co-author of the publications describing the EMERGE assay and declares no financial competing interest related to this method. Jean-Baptiste Fini declares no competing interests. None of the authors had any role in the preparation of the article commented upon.

## CRediT authorship contribution statement

**Gregory F. Lemkine:** Conceptualization, Writing – original draft, Writing – review & editing. **François Brion:** Conceptualization, Writing – original draft, Writing – review & editing. **Xavier Coumoul:** Conceptualization, Writing – review & editing, Writing – original draft. **Jean-Baptiste Fini:** Conceptualization, Writing – original draft, Writing – review & editing.

## Data Availability

No data was used for the research described in the article.

## References

[bib0001] Brion F., Le Page Y., Piccini B., Cardoso O., Tong S.-K., Chung B.-C., Kah O. (2012). Screening estrogenic activities of chemicals or mixtures in vivo using transgenic (cyp19a1b-GFP) zebrafish embryos. PLoS One..

[bib0002] Christou M., Georgantzopoulou A., Hultman M., Song Y., Wennberg A.C., Mentzel S. (2026). The rise of new approach methodologies (NAMs) for environmental safety assessment of chemicals: a systematic review of the current status, challenges and strategies for broader adoption of NAMs. NAM J..

[bib0003] Du Pasquier D., Salinier B., Coady K.K., Jones A., Körner O., LaRocca J., Lemkine G., Robin-Duchesne B., Weltje L., Wheeler J.R., Lagadic L. (2024). How the Xenopus eleutheroembryonic thyroid assay compares to the amphibian metamorphosis assay for detecting thyroid active chemicals. Regul. Toxicol. Pharmacol..

[bib0004] Du Pasquier D., Tindall A., Weltje L., Lagadic L., Lemkine G. (2025). The Xenopus eleutheroembryo thyroid assay (XETA), a new approach methodology (NAM) for the regulatory assessment of thyroid activity of chemicals. Environ. Toxicol. Chem..

[bib0005] Erradhouani C., Piccini B., Maillot-Marechal E., Aït-Aïssa S., Balaguer P., Coumoul X., Brion F. (2025). New insights into the regulation of cyp3a65 expression in transgenic tg(cyp3a65:GFP) zebrafish embryos. Aquat. Toxicol..

[bib0006] Erradhouani C., Geffroy F., Piccini B., Hinfray N., Chadili E., Balaguer P., Sohm F., Aït-Aïssa S., Coumoul X., Brion F. (2025). A new alternative method using cyp3a65 expression in transgenic zebrafish embryos to assess metabolic endocrine-disrupting chemicals in the intestine. Environ. Int..

[bib0007] European Commission (2026). Communication from the commission: roadmap towards phasing out animal testing for chemical safety assessments. C(2026) 3497 final, 1 June 2026. https://single-market-economy.ec.europa.eu/sectors/chemicals/reach/roadmap-towards-phasing-out-animal-testing_en.

[bib0008] European Commission, accessed 27 August 2026 (2026). Commission staff working document SWD(2026) 144 final, accompanying the communication "roadmap towards phasing out animal testing for chemical safety assessments", 1 June 2026. https://single-market-economy.ec.europa.eu/document/download/4afe05f9-35ec-46b7-83bd-a9ae95111916_en.

[bib0009] Fini J.-B., Le Mével S., Turque N., Palmier K., Zalko D., Cravedi J.-P., Demeneix B.A. (2007). An in vivo multiwell-based fluorescent screen for monitoring vertebrate thyroid hormone disruption. Environ. Sci. Technol..

[bib0010] Hinfray N., Tebby C., Piccini B., Bourgine G., Aït-Aïssa S., Porcher J.-M., Pakdel F., Brion F. (2018). Mixture concentration-response modeling reveals antagonistic effects of estradiol and genistein in combination on brain aromatase gene (cyp19a1b) in zebrafish. Int. J. Mol. Sci..

[bib0011] Jaka O., Iturria I., Martí C., Hurtado de Mendoza J., Mazón-Moya M.J., Rummel C., Weiner A.M.J., Muriana A. (2023). Screening for chemicals with thyroid hormone-disrupting effects using zebrafish embryo. Reprod. Toxicol..

[bib0012] Mitchell C.A., Burden N., Bonnell M., Hecker M., Hutchinson T.H., Jagla M., LaLone C.A., Lagadic L., Lynn S.G., Shore B., Song Y., Vliet S.M., Wheeler J.R., Embry M.R. (2023). New approach methodologies for the endocrine activity toolbox: environmental assessment for fish and amphibians. Environ. Toxicol. Chem..

[bib0013] OECD (2019).

[bib0014] OECD (2021).

[bib0015] OECD (2022).

[bib0016] OECD (2024).

[bib0017] Pesce E., Garde M., Rigolet M., Tindall A.J., Lemkine G.F., Baumann L.A., Sachs L.M., Du Pasquier D. (2024). A novel transgenic model to study thyroid axis activity in early life stage medaka. Environ. Sci. Technol..

[bib0018] Pesce E., Benitez-Gonzalez J., Tindall A.J., Lemkine G.F., Robin-Duchesne B., Sachs L.M., Du Pasquier D. (2024). Testing the sensitivity of the medaka transgenic eleuthero-embryonic THYroid-specific assay (TETHYS) to different mechanisms of action. Aquat. Toxicol..

[bib0019] Petersen K., Fetter E., Kah O., Brion F., Scholz S., Tollefsen K.E. (2013). Transgenic (cyp19a1b-GFP) zebrafish embryos as a tool for assessing combined effects of oestrogenic chemicals. Aquat. Toxicol..

[bib0020] Sébillot A., Damdimopoulou P., Ogino Y., Spirhanzlova P., Miyagawa S., Du Pasquier D., Mouatassim N., Iguchi T., Lemkine G.F., Demeneix B.A., Tindall A.J. (2014). Rapid fluorescent detection of (anti)androgens with spiggin-gfp medaka. Environ. Sci. Technol..

[bib0021] Spirhanzlova P., Leleu M., Sébillot A., Lemkine G.F., Iguchi T., Demeneix B.A., Tindall A.J. (2016). Oestrogen reporter transgenic medaka for non-invasive evaluation of aromatase activity. Comp. Biochem. Physiol. C Toxicol. Pharmacol..

[bib0022] Tong S.-K., Mouriec K., Kuo M.-W., Pellegrini E., Gueguen M.-M., Brion F., Kah O., Chung B.-C. (2009). A cyp19a1b-gfp (aromatase B) transgenic zebrafish line that expresses GFP in radial glial cells. Genesis.

